# Neuroticism, resilience, and social support: correlates of severe anxiety among hospital workers during the COVID-19 pandemic in Nigeria and Botswana

**DOI:** 10.1186/s12913-021-06358-8

**Published:** 2021-06-01

**Authors:** Anthony A. Olashore, Oluyemi O. Akanni, Kehinde O. Oderinde

**Affiliations:** 1 Department of Psychiatry, University of Botswana, Gaborone, Botswana; 2 Clinical Services, Federal Neuro-Psychiatric Hospital, Benin City, Nigeria; 3 Department of Mental Health, University of Benin Teaching Hospital, Benin City, Nigeria

**Keywords:** Anxiety, Botswana, COVID-19 pandemic, Healthcare workers, Neuroticism, Nigeria, Resilience

## Abstract

**Background:**

The role of healthcare workers (HCWs) during the COVID-19 pandemic may make them more susceptible to anxiety than the general population. This study aimed to determine the prevalence of anxiety and evaluate the potential effects of resilience, neuroticism, social support, and other sociodemographic factors on anxiety among HCWs from two African countries.

**Methods:**

A cross-sectional survey of 373 HCWs was conducted in Botswana and Nigeria, using an anxiety rating scale, neuroticism subscale of Big Five Inventory, Oslo social support scale, and Resilience Scale. Data collection was done between May 1 and September 30, 2020.

**Results:**

The participants’ mean age (SD) was 38.42 (8.10) years, and 65.1% were females. Forty-nine (13.1%) of the HCWs reported clinical anxiety. In the final model of hierarchical multiple regression, neuroticism (B = 0.51, t = 10.59, p = *p* < 0.01), resilience (B = 0.34, t = − 7.11, *p* < 0.01), and social support (B = 0.079, t = − 2.11, *p* = 0.035) were associated with severe anxiety, after controlling for the significant sociodemographic factors.

**Conclusions:**

Severe anxiety exists among HCWs in Africa, although the rate was lower than reported elsewhere. Neuroticism, resilience, and social support may be vital targets for psychological intervention in a pandemic as COVID-19; thus, their roles should be further explored.

## Background

Pandemic, such as COVID-19, threatens the physical and psychological well-being of people [1]. The health care workers (HCWs), who are directly involved in the diagnosis, treatment, and care of patients with COVID-19, may be at risk of developing mental health symptoms such as anxiety [2]. While some anxiety levels are necessary for performance, the high or clinical level may be highly disruptive as it reduces productivity and health care delivery by the HCWs, especially during this pandemic when they are highly needed [3, 4].

Anxiety symptoms have been reported in about 23% of HCWs during this pandemic [5]. A rate as high as 45% was reported among this group in China [6]. Information regarding the prevalence in Africa still lacks at the time of writing; however, the continent is expected to be the most vulnerable to the psychological impact of COVID-19 [7].

The ever-increasing number of confirmed and suspected cases, overwhelming workload, depletion of personal protective equipment (PPE), widespread media coverage, and fear of acquiring the infection or transmitting it to their family members have been observed to contribute to the high rate of anxiety in the HCWs [8, 9]. Besides, some authors have identified the role of sociodemographic factors like advanced age and living alone in the development of anxiety during COVID-19 [9]. Beyond these factors, research has shown that personal differences in temperament predict psychological well-being during the crisis [10, 11]. Neuroticism is one of these basic personality traits [12], which entails negative emotionality and represents the degree to which a person experiences the world as distressing, threatening, and unsafe [12]. Individuals with high neuroticism perceive the world as threatening and may become emotionally distressed [12]. Affected individuals are thus likely to develop negative emotions or anxiety at a time like this.

Furthermore, social support levels have been closely related to the occurrence of anxiety [13]. Low social support levels can increase an individual’s vulnerability to psychological distress, such as anxiety symptoms when exposed to stress [13, 14]. For example, a study conducted in China among adolescents during this pandemic reported a higher prevalence of anxiety in those who received medium and low social support levels when compared to those with high social support [15]. In contrast, supportive supervision and peer support networks were protective in a study conducted during an epidemic in West Africa [16]. One significant way social support protects against anxiety or other psychological disorders during stressful events is by building resilience [17].

Resilience occurs when mental processes and behaviours are used to promote psychological assets and prevent possible adverse effects of stressful events such as the COVID-19 pandemic [13]. In support of this, a recent study conducted among physicians during this pandemic reported an association between reduced anxiety and resilience rate [18].

Against this backdrop, it is reasonable to hypothesize that high levels of social support and resilience and low levels of neuroticism could aid the mental health of HCWs during this COVID-19 pandemic and could be used in formulating preventive measures. Regrettably, most of these published reports on anxiety and its associated factors were conducted outside Africa [5, 6] and may not apply in our setting. The possible influence of the cultural difference in psychological response to stressful events [19, 20] makes it pertinent to repeat this research in our setting. Hence, we aimed to determine the prevalence of anxiety and evaluate the potential effects of resilience, neuroticism, social support, and other sociodemographic factors on anxiety among HCWs from two African countries. We hoped that establishing the rate of anxiety and factors that can worsen or ameliorate it will help formulate a locally adaptive and inexpensive preventive measure.

## Methods

Our study design was cross-sectional, and it involved three centres from two African countries: Nigeria and Botswana. Two centres were selected from south-south Nigeria: The Federal Neuropsychiatric Hospital (FNPH) and the University of Benin Teaching Hospital (UBTH), Benin-City. The FNPH is a 270-bed facility that provides in-patient and out-patient care and emergency services to mentally ill persons primarily across the region of Nigeria. The UBTH has 600 beds for in-patient care and caters to both medical and surgical conditions. It has a daily turnout of about 350 patients and receives referrals from other neighbouring health facilities. The third centre was selected from Botswana, which is the Sbrana Psychiatric Hospital (SPH). It is the only referral mental hospital in the country, and it is in the south-western part. It has a capacity of 300 beds dedicated mainly to adult mental health care. Data collection was done between May 1 and September 30, 2020.

Approval for the study was obtained from the Research and Ethics Committee of the three Hospitals and written informed consent from the participants to qualify for recruitment into the study. The minimum sample size was determined using the formula *z*^2^*pq*/*d*^2^, (*z* – the standard normal distribution, set here at 1.96, *p* – the proportion of the target population estimated to have a particular characteristic, *q* = 1.0 *− p* and *d* = degree of accuracy desired, here is 0.05) [21]. The minimum sample size of 315 was calculated using a recent study in which moderate to severe anxiety prevalence among health workers was 28.8% [22]. However, the final sample of 373 collected during questionnaire administration exceeded the minimum. All staff members of the hospitals present during questionnaire distribution were interviewed.

The selection criteria consisted of the staff with close contact with patients and were working in the selected health facilities: physicians, nurses, pharmacists, laboratory scientists and technicians, record officers, and health attendants. All the consenting personnel listed above that were available during the study period were recruited. The support staff excluded were the security guards and all the administrative staff because they have little or no contact with patients. Members of staff who did not consent were also excluded. The following tools were used for this study**:**

- **Socio-demographic Data Collection Sheet** was explicitly designed to inquire about the participant's sociodemographic characteristics such as gender, age, marital status, education level, profession, and religion.
- **The Anxiety rating scale** [23] was used to measure health workers’ anxiety levels. The questionnaire contains ten questions based on feelings of anxiety concerning COVID-19 in the previous 7 days. Some of the items are: ‘I feel tense, nervous, restless, or agitated,’ ‘I wish I knew a way to make myself more relaxed’ and ‘I worry about bad things that might happen to me or those I care about.’ Each item has five grades of anxiety from ‘never’ to ‘always,’ with scores ranging between 0 and 4. The total score is derived from the addition of all the points from each item. A total score ‘0 to 8 point’ is regarded as minimal anxiety, ‘8 to 16’ points, mild anxiety, ‘17 to 24’ points, moderate anxiety, ‘25 to 32’ points high anxiety (Warning Level requiring clinical attention), and ‘33 to 40’ points extremely high anxiety. In the present study, a cut-off point of 25 was adopted to indicate a high level of anxiety requiring clinical intervention [24]. The Cronbach alpha for the instrument in this study was 0.96.
- **The neuroticism subscale of the 44-item Big Five Inventory** [25] contains seven items, and it is a self-report inventory designed to measure the neuroticism personality trait reliably. The personality trait has been identified reliably in different cultures and countries, including Africa [26], and modified in an earlier study to make it more culture-sensitive [27]. It has the advantage of being relatively quick to complete because the items consist of short sentences like: ‘I see myself as someone who can be tense,’ ‘I see myself as someone who can be moody,’ and ‘I see myself as someone who worries a lot.’ The items are rated on a 5-point scale ranging from 1 (disagree strongly) to 5 (agree strongly). Higher scores indicate higher neuroticism. A Cronbach alpha of 0.89 was obtained in the present study.
- **The Oslo 3-item Social Support Scale (OSS-3)** [28] provides a brief measure of social support covering different social support fields by measuring the number of people the respondent feels close to, the interest and concern showed by others and the ease of obtaining practical help. It has been found to have good psychometric properties in the Nigerian population, in addition to its brevity and clarity [29]. The OSS-3 scores were added together, and high scores indicate strong support. Internal consistency of 0.81 was obtained in this study.
- **The 14-Item Resilience Scale** [30] was used to identify the degree of resilience on personal competence and acceptance of self and life (e.g., “I feel that I can handle many things at a time”; “my life has meaning”). The Resilience Scale generally appears to have the widest use across different age groups because of its readability. The scale has been applied to some populations in Nigeria [31]. It takes about 3–5 min to complete. Responses are made on a 7-point scale (1 to 7), and higher scores indicate greater resilience. A Cronbach reliability of 0.94 was obtained in the current study.

Data collected were analyzed using the Statistical Package for Social Sciences (SPSS) version 22. All the sociodemographic variables except participants’ profession and center were dichotomized to enable a sufficient number of participants for analysis, e.g., the marital status was categorized into married and unmarried (single, widow, divorced). The variable ‘profession’ was categorized into doctors, nurses, and others. Descriptive statistics were used, such as frequencies and mean. Inferential statistics included Chi-square, T-test, and hierarchical multiple regression. A Chi-square test was used to test the association between anxiety and other variables like gender, marital status, religion, profession, and country. A t-test was used to compare the differences in the mean score of age, resilience, neuroticism, social support, and anxiety. Two-step hierarchical multiple regression was carried out. In the first step, significant demographic factors such as age, country, and religion were entered to control for their effects. Thereafter, the remaining significant variables, such as neuroticism, resilience, and social support, were entered at Step 2 to determine their independent effects on anxiety. The level of significance was set at a *p*-value of less than 0.05.

## Results

Out of the 402 questionnaires distributed, 373 questionnaires properly filled were analyzed, giving a response rate of 92.8%. The respondents' mean age (SD) was 38.42 (8.10) years, and about 65% (242) were females). Two-thirds of the participants were from Nigeria. Nurses formed more than half (57.6%) of the workforce sampled, while the married to the unmarried ratio was 2:1. The majority of the workers were Christians (89.2%) and had at least tertiary educational attainment (94.0%) (Table 1).

**Table 1:** Socio-demographic characteristics of participants

Variables	Characteristics	No of participants (%)
**Gender**^**a**^	Male	130 (34.9)
	Female	242 (65.1)
**Country**	Nigeria	252 (67.6)
	Botswana	121 (32.4)
**Marital status**	Unmarried	124 (33.2)
	Married	249 (66.8)
**Educational qualification**^**a**^	Secondary & below	22 (6.0)
	Tertiary & above	347 (94.0)
**Religion**^**a**^	Christianity	330 (89.2)
	Others^b^	40 (10.8)
**Profession**	Doctor	41 (11.0)
	Nurse	215 (57.6)
	Others^c^	117 (31.4)
**Centre**	UBTH	123 (33.0)
	FNPH	129 (34.6)
	SPH	121 (32.4)
**Anxiety**^**a**^	None to mild	313 (86.5)
	Severe	49 (13.5)

Mean age is 38.42 years

*FNPH*: Federal Neuropsychiatric Hospital and the

*UBTH*: University of Benin Teaching Hospital

*SPH*: Sbrana Psychiatric Hospital

^a^ Figure did not add up to 373 because of missing data

^b^ Others are Islam, traditional religion

^c^ Others are clinical psychologist, occupational therapist, medical record officer, etc

Applying a cut-off point of 25 on the Anxiety Rating Scale, 49 (13.5%) of the workers reported severely elevated anxiety (Table 1). There were significant associations of severe anxiety with the country Nigeria (X^2^ = 5.22, *p* = 0.02), the Christian religion (X^2^ = 5.34, *p* = 0.02), older age (t = − 2.89, *p* = 0.01), lower resilience (t = 49.80, *p* < 0.01), higher neuroticism (t = − 17.15, *p* < 0.01) and lower social support (t = 4.93, *p* < 0.01) (Tables 2 & 3).

**Table 2:** Association between anxiety problem and participants’ characteristics

Variables	Anxiety problem		X^**2**^	***p***-value
	None-mild (%)	Severe (%)		
**Gender**				
Male	108 (85.0)	19 (15.0)	0.32	0.57
Female	204 (87.2)	30 (12.8)		
**Country**				
Nigeria	204 (83.6)	40 (16.4)	5.22	0.02*
Botswana	109 (92.4)	9 (7.6)		
**Marital status**				
Married	205 (84.4)	38 (15.6)	2.79	0.10
Others	108 (90.8)	11 (9.2)		
Educational status**				
Sec & below	18 (81.8)	4 (18.2)	0.40	0.52
Ter & above	291 (86.6)	45 (13.4)		
**Religion**				
Christianity	281 (87.8)	39 (12.2)	5.34	0.02*
Others	29 (74.4)	10 (25.6)		
**Profession**				
Doctor	34 (85.0)	6 (15.0)	4.56	0.10
Nurse	189 (89.6)	22 (10.4)		
Others	90 (81.1)	21 (18.9)		

*significant *p*-value ** Fisher’s exact test

**Table 3:** Mean difference between severe and mild/none cases of anxiety

Variables	Anxiety	N	Mean	SD	df	t	P
**Age**					353	−2.89	0.01*
	None-mild	306	37.99	8.26			
	Severe	49	40.71	5.71			
**Social support**					359	4.93	< 0.001*
	None-mild	312	11.19	2.47			
	Severe	49	6.27	2.53			
**Resilience**					348	49.80	< 0.001*
	None-mild	301	82.29	13.06			
	Severe	49	32.49	22.63			
**Neuroticism**					356	−17.15	< 0.001*
	None-mild	310	6.77	5.65			
	Severe	48	23.92	5.57			

*Significant *p*-value

Hierarchical multiple regression was used to assess the effect of neuroticism, resilience, and social support on anxiety levels after controlling for the influence of the significant demographic factors. Age, country, and religion were entered in Step 1 and found to explain 1.1% of the variance in anxiety. After the entry of neuroticism, resilience, and social support at Step 2, the total variance explained by the model was 77.0%, F (6, 331) = 184.32, *p* < .001. The combination of neuroticism, resilience, and social support explained an additional 75.8% of the variance in anxiety after controlling for age, country, and religion, R squared change = .758, F change (3, 331) = 363.30, *p* < .001.

In the final model, only the neuroticism (B = 0.51, t = 10.59, p = *p* < 0.01), resilience (B = 0.34, t = − 7.11, *p* < 0.01), and social support (B = 0.079, t = − 2.11, *p* = 0.035) were statistically significant, with the neuroticism recording the highest beta value (Table 4).

**Table 4:** Hierarchical Regression Analysis of variables associated with anxiety

	Step 1			Step 2		
Variable	B	t	*p*-value	B	t	*p*-value
Age	0.068	1.213	0.226	0.042	1.532	0.127
Country	0.040	0.717	0.474	−0.026	−0.903	0.367
Religion	0.082	1.505	0.133	0.049	1.855	0.064
Social support				−0.079	−2.114	0.035*
Resilience				−0.344	−7.107	< 0.001*
Neuroticism				0.516	10.589	< 0.001*
R^2^ change	0.01			0.76		
df	334			331		
F change	1.26			363.30*		

*Significant *p*-value

## Discussion

The present study sought to access anxiety and its relationship with psychosocial factors among HCWs using samples from two African countries. The prevalence of severely elevated anxiety in the HCWs according to the Anxiety Rating Scale was 13.5%. Several studies have revealed varying rates regarding the psychological reaction to COVID-19 during this period [5, 6]. The pooled prevalence of anxiety from a systematic review of studies conducted outside Africa was 26%, and it ranged from 18 to 32% [32]. The rate reported in our study falls below this range and may be due to some factors.

The utilization of culturally adaptive coping strategies and the cultural effect of psychological symptoms expression in African settings may have played a role in the reported rate of anxiety in the present study. For example, Africans show greater inhibition and emotional defensiveness than Caucasians and Europeans [33, 34]. While they can be maladaptive, these repressive coping styles and active processing of emotions have been linked to adaptive well-being and positive health reports [35, 36]. A study has shown that Africans report less anxiety and greater perceived control over anxiety-provoking events and their reactions to these events [19]. Moreover, Africans are more likely to report anxiety as somatic or pay more attention to physical symptoms of anxiety than cognitive complaints [19, 20]. For example, “heat” or “peppery” sensations in the body, especially the head and feet, are typical in the presentation of anxiety in Africa [20]. These culturally specific symptoms are not always captured by the most available screening tools and may require different diagnostic strategies. A more culturally adapted tool may better reflect the anxiety level in this population and should be considered in future research.

The study participants’ composition may have played another enormous role in the anxiety levels recorded in the current study. About two-thirds of our participants were mental health staff who maybe well-equipped in managing anxiety. Also, it is believed that these countries have experienced a more devastating effect of epidemics in the past. These include HIV infection in Botswana and Ebola in West Africa. These may have increased their ability to cope with stress. Furthermore, Africa’s death rate regarding COVID-19 is less than what has been reported in other continents [7]; thus, uncertainty about death resulting from the infection is less. However, the presence of anxiety disorder in our population suggests the need for interventions designed to reduce this disorder and improve their ability to cope with the enormous challenges of care for patients during this pandemic.

Additionally, this study explored the factors that determine who develops anxiety symptoms in our sample. Sociodemographic factors such as age, country, and religion were found to be related to anxiety symptoms, in addition to neuroticism, resilience, and social support. When we controlled for the influence of these significant sociodemographic factors, it became apparent that neuroticism, resilience, and social support explained about 76% of the variance in anxiety. This finding suggests that these psychosocial factors play a huge role in determining who develops anxiety disorder in our population and deserves to be fully understood to prevent the COVID-19 pandemic’s psychological impact.

High neuroticism is one of the Big Five personality traits frequently found in anxious patients [12]. It is associated with the processing of fear-inducing emotion and increases anxiety vulnerability [12]. In the present study, those who had high neuroticism scores complained of severe anxiety symptoms, as previously documented [37, 38]. As hitherto alluded to, persons with high neuroticism scores often pay more attention to COVID-19 related information and are anxious about its sequels on their health [38]. This consequent negative emotion tends to impair judgment, productivity, and health care delivery [10]. Our findings further buttress the role of personality traits as a predictor of psychopathology in HCWs during pandemics and should be addressed to enhance the efficiency of health care workers. 

We found an inverse correlation between resilience and anxiety in our study. Psychological resilience characterizes a method of adapting well in the face of stress-provoking events such as the COVID-19 pandemic, and it is a function of high-quality positive social support [39]. Studies conducted during this pandemic have revealed high anxiety symptoms among participants with low resilience and a lower rate among physicians with high resilience scores [14, 16]. Individuals with low resilience tend to have worse mental health outcomes such as anxiety and depression due to low adaptability to stressful life events as COVID-19. Psychological resilience has the potential ability to protect individuals during crises and help cope with disasters [13]. Hence, mental resilience may be a vital target for psychological intervention in a pandemic as COVID-19.

The participants who reported having low perceived social support complained of more severe anxiety symptoms in this study. Perceived social support may be central to anxiety regardless of gender, age, race, and religious affiliations and may have important implications for developing anxiety interventions. Dour and colleagues [39], in a study conducted in a primary health care setting, reported a strong positive relationship between perceived social support and anxiety after controlling for the mediating effect of gender, race, and age. Roohafza and colleagues [14] also reported that perceived social support is protective against anxiety symptoms. Social support may be one of the coping tools that keep HCWs in Africa functioning in the absence of adequate health care infrastructure, especially during epidemics [16], and may also account for the low rate of anxiety symptoms in our sample.

Over the years, Africans have annexed a close-knit family system’s social benefit, especially in emotional resource sharing as an inexpensive way of coping during the crisis. This was affirmed by a West African study, which underscores the role of peer and family support in building resilience against mental exhaustion during the Ebola epidemic [16]. Even though the present study did not explore how these variables interrelate with themselves, previous studies have shown that social support plays a huge role in addressing low resilience and high neuroticism [17]. Social support confers resilience to individuals with high neuroticism in stressful events [13] and should be offered to HCWs as a preventive measure against psychological reactions such as anxiety during pandemics. Future studies should focus on how social support can be integrated into the preventive package against psychological distress during a pandemic. Besides, other ways of addressing neuroticism and building resilience in African settings should be explored and put in place to further prevent anxiety among the HCWs.

Finally, there was no professional difference in the level of anxiety reported. This is contrary to the higher prevalence of anxiety previously reported among nurses when compared to other hospital workers in other climes [5]. Nurses are more prone to stress and exhaustion due to the shift-duty nature of their work and prolonged contact with patients. However, it is interesting to note that most African studies are in keeping with our finding of a non-significant relationship of the HCWs' profession with anxiety during COVID-19 [40–42]. The reason for this is uncertain and will require further investigation.

Nevertheless, our study has some limitations which must be discussed. Due to our study’s cross-sectional nature, the causal associations between sociodemographic factors and anxiety could not be deduced, so we could not conclude on this. Although our sample size may be small for a multisite study, it is adequate to provide statistical power to detect significant associations. This, we believe, could limit the generalizability of our findings to the entire population of HCWs in both countries. The use of non-culturally adapted tools and perceived social support constructs limit the interpretation of our findings. The use of self-reported questionnaires may have resulted in underreporting or overreporting of symptoms. This limitation and the lack of proportional allocation in sample selection may have distorted the reported prevalence.

Finally, our study accounted for about 76% of the variance in anxiety among HCWs during the COVID-19 pandemic. This finding suggests that other factors related to its development were not investigated and should be explored in future research. Also, a cohort study in a carefully defined group of HCWs and the use of culturally adapted tools may be necessary to reveal a clearer picture of anxiety in this population.

## Conclusions

Severe anxiety exists among HCWs who participated in this study. Even though the rate was lower than reported elsewhere, there is a need for interventions aiming to reduce anxiety and improve their ability to cope with the enormous challenges of care for patients during this pandemic. Our study also found that high neuroticism and low resilience predict the development of anxiety symptoms in HCWs during COVID-19, while social support, when available, protects against this psychological disorder. These factors may be vital targets for psychological intervention in a pandemic as COVID-19; thus, their roles should be further explored.

## Data Availability

The datasets used and analysed during the current study are available from the corresponding author on reasonable request.

## Abbreviations

HCWs: Health Care Workers
FNPH: Federal Neuropsychiatric Hospital and the
UBTH: University of Benin Teaching Hospital
IRB: Independent Review Board
SPH: Sbrana Psychiatric Hospital
